# Comparison of long-term outcome between muscle sparing and non-muscle sparing surgical techniques in rib plating

**DOI:** 10.1007/s00068-025-02881-z

**Published:** 2025-05-20

**Authors:** Erik Öberg Westin, Monika Fagevik Olsén, Eva-Corina Caragounis

**Affiliations:** 1 Department of Surgery, Institute of Clinical Sciences, Sahlgrenska University Hospital, Per Dubbsgatan 15, 413 45 Gothenburg, SE Sweden; 2 Department of Physical Therapy, Institute of Neuroscience and Physiology, Sahlgrenska Academy, University of Gothenburg, Gothenburg, Sweden; 3 Department of Physiotherapy, Faculty of Health Sciences, University of the Witwatersrand, Johannesburg, South Africa; 4 Department of Surgery, Region Västra Götaland, Sahlgrenska University Hospital, Gothenburg, Sweden; 5 Region Västra Götaland, Department of Occupational Therapy and Physiotherapy, Sahlgrenska University Hospital, Gothenburg, Sweden

**Keywords:** Chest wall injury, SSRF, Muscle-sparing, Surgical technique, Long-term outcome

## Abstract

**Purpose:**

Surgical stabilization of rib fractures is an internationally established method for treating traumatic chest wall injuries. Few studies have compared the various surgical methods used. The aim of this study was to examine how different surgical methods affect long-term outcomes.

**Method:**

This is a study of prospectively included patients with flail chest undergoing muscle-sparing chest wall surgery (*n* = 40) who were compared to historical controls who underwent conventional surgery with non-muscle-sparing incisions and thoracotomy (*n* = 38). The cohorts differed regarding injury severity, smoking habits and number of ribs operated. This was adjusted for using multiple linear regression. The primary endpoint was lung function, secondary endpoints were respiratory muscle strength, respiratory movement, physical function, physical activity, and quality of life (QoL) after six and 12 months.

**Results:**

Seventy-eight patients (67.9% men and 32.1% women) with a mean age of 63.6 ± 14.0 years were included. The predicted forced vital capacity (FVC) was 92.3 ± 14.3% vs. 85.0 ± 15.3% after 12 months (*p* = 0.037) in the muscle-sparing and conventional surgery cohorts, respectively. Shoulder movement (Boström Index 59 vs. 56, *p* = 0.007) and lateral flexion (16.1 vs. 11.4 cm, *p* = 0.004) were significantly better in the muscle-sparing surgery patients than the conventional surgery patients after one year. No significant differences were found in respiratory muscle strength, respiratory movement, physical activity, or QoL.

**Conclusion:**

Patients who undergo muscle-sparing surgery for chest wall injury have better long-term lung function, shoulder movement, and thoracic movement than patients who undergo conventional surgery. No difference was found between the groups concerning self-reported outcome.

Registered in www.clinicaltrials.gov at 2020–12-18 with the ID NCT04710602.

**Supplementary Information:**

The online version contains supplementary material available at 10.1007/s00068-025-02881-z.

## Background

Surgical stabilization of rib fractures (SSRF) has become an internationally established treatment for traumatic chest wall injuries. Several randomized studies have shown that surgery is superior to nonoperative treatment for flail chest injuries [1–5]. Surgery for flail chest injuries has been shown to decrease the length of stay (LOS) in the hospital [2, 5], duration on a ventilator [1, 3–5], and the LOS in an intensive care unit (ICU) [1–5]. Surgery has also been shown to reduce complications associated with flail chest injury, such as pneumonia [1, 4–6] and the incidence of tracheostomy [1, 3], compared to nonoperative treatment.

Different techniques and materials are used for SSRF, and no clear consensus has been reached on surgical technique. Wide incisions, with or without concomitant thoracotomy, have been suggested to ensure wide exposure and control of intrathoracic injuries [7–9]. Some authors have outlined an evolution in the surgical technique from large, non-muscle-sparing incisions to a less invasive approach [10, 11]. A muscle-sparing open technique to minimize injuries to soft tissue was described in the first randomized study on SSRF [1]. Similar methods have been utilized by other authors [11, 12], occasionally with thoracoscopic guidance, which has the added benefit of being able to address intrathoracic pathology [13, 14]. Ultrasound has been used to identify fracture locations preoperatively [15], whereas others have tried a pure thoracoscopic approach [16–18].

Few studies have compared the long-term outcomes of different surgical techniques for rib plating. A retrospective study showed shorter time on a ventilator, shorter ICU LOS, shorter hospital LOS, and a decreased incidence of pneumonia after using a muscle-sparing open approach versus an open procedure with large, non-muscle-sparing incisions and thoracotomy [19]. A prospective study from 2023 reported shorter hospital LOS with a thoracoscopic technique compared to open rib plating [20], and a retrospective study from 2023 showed less postoperative pain with an intrathoracic technique [21]. None of these studies specified whether the open control method was muscle-sparing. To the best of our knowledge, no prospective study has evaluated long-term outcomes with different surgical techniques for chest wall injury. Due to this lack of comparative data, uncertainty exists as to whether the surgical technique influences long-term results.

Our hypothesis is that a muscle-sparing surgical technique for chest wall injuries improves long-term outcomes regarding lung function, mobility, physical function, disability, and quality of life (QoL).

## Methods

A single-institution study with prospectively included patients with historical controls was performed to compare the long-term outcome of chest wall surgery for flail chest with different surgical techniques. The primary endpoint of the study was lung function. The secondary endpoints were respiratory muscle strength, respiratory movement, physical function, physical activity, shoulder mobility, and QoL. As no previous studies had compared the long-term results of different surgical techniques for chest wall injuries when this study was initiated, the sample size was estimated from a power analysis based on a preliminary analysis that found significant differences in lung function between 18 patients treated with muscle-sparing SSRF and 34 patients treated with non-muscle-sparing SSRF with concomitant thoracotomy [6]. To calculate the sample size for the trial, the power analysis was based on a mean difference in the predicted forced vital capacity (FVC) of 14%, a standard deviation of 17%, a power of 0.80, and an alpha of 0.05. According to this analysis, 23 patients needed to be included in each surgical group to achieve significant results in the predicted FVC. We elected to include 50 patients to account for patients lost to follow-up and to determine significant differences in secondary outcome measures. The study was approved by the Swedish Research Ethics Committee (Dnr 2020–04509). The study was registered at www.clinicaltrials.gov before inclusion commenced (NCT04710602). The STROBE guideline was used to ensure proper reporting.

Inclusion criteria were adult (age ≥ 18 years) patients with flail chest who underwent chest wall surgery with plate fixation for trauma during the acute care period (≤ 14 days). Flail chest was defined as at least two rib fractures on at least three consecutive ribs [22], sternal flail (i.e., at least two bilaterally fractured ribs with two concomitant fractures in the sternum, or a vertical and horizontal sternum fracture combined with two unilateral rib fractures) [23]. A flail chest was also deemed to be present with clinically apparent paradoxical chest wall movement [22]. Exclusion criteria were severe traumatic brain injury with abbreviated injury score (AIS) > 3 [22], spinal injuries, known neurological or musculoskeletal disease affecting movement of the rib cage, and lung resection performed during SSRF. Oral and written informed consent was obtained from all participants prior to their inclusion in their respective cohorts. Patients were seen by a surgeon and physiotherapist six and 12 months after surgery.

A consecutive series of adult (≥ 18) patients with flail chest undergoing muscle-sparing chest wall surgery (hereafter, muscle-sparing surgery) during 2020–2022 were invited to participate in the study until we reached the predefined number of 50. The procedure was performed using an open, muscle-sparing approach without thoracotomy or thoracoscopy. The plan for each procedure was a short incision length and non-traumatizing handling of tissues with no division of muscle fibers, minimizing injury to the nerves and blood vessels. Careful preoperative planning where we combined computed tomography (CT) imaging with anatomical landmarks, as the scapula, mammilla, and spine, was used to achieve optimal incisions when fractures were not palpable. Undermining of the skin facilitated movement of the incision over the chest wall, making the repair of several ribs possible through small incisions. Multiple incisions were used when deemed necessary to repair a larger number of fractures.

As a control, we used a cohort of 57 patients who participated in earlier prospective studies and underwent surgery for flail chest injuries using a non-muscle-sparing technique with concomitant thoracotomy between 2010 and 2017 (hereafter, conventional surgery) and underwent clinical follow-up until 2018 [6, 24]. These patients were operated on at our institution, and the co-authors were involved in these studies and had access to raw patient data. The transition from non-muscle-sparing SSRF with concomitant thoracotomy to muscle-sparing SSRF without thoracotomy was a gradual process that coincided with a new surgeon joining the team but was also based on experience with unnecessary thoracotomies and the idea of reducing surgical trauma to the chest wall.

Conventional surgery was performed with a non-muscle-sparing thoracotomy for ease of access, control of drilling, management of intrathoracic injuries, and pleural lavage. Patients were ventilated with a double lumen endotracheal tube to deflate the lung. Rather than making multiple incisions, the incisions were large and extended. Careful preoperative planning combined CT imaging and anatomical landmarks to ensure the incision was made at the right level [8].

Chest drains (28–32 Fr) remained in place after both procedures. For both procedures, the patient was placed in the position best suited for the planned repair, most often the side position. Repairs for both techniques were made using the MatrixRIB™ (DePuy Synthes, Westchester, USA) fixation system. The aim of surgery was to stabilize the flail segment, especially ribs 4–10, not to fix all fractures. Surgeries were performed by five different surgeons. Follow-up was completed by two surgeons and two specialized physiotherapists.

Baseline data were collected from patient records and the Swedish trauma registry (SweTrau). Collected data included age; sex; body mass index (BMI); smoking status; comorbidities, such as chronic obstructive pulmonary disease (COPD), asthma, lung emphysema, and diabetes; the AIS score [22] for head and thorax; Injury Severity Score (ISS) [25] and New Injury Severity Score (NISS) [26]; number of fractured ribs; presence of pneumothorax and/or hemothorax; day of surgery; number of ribs operated; and percentage of fractured ribs operated.

The subsequent follow-up was performed on all patients six and 12 months after surgery. All tests were performed in the same way in both groups with the same or comparable instruments by the same two physiotherapists. Standardized lung function tests were performed [27] with spirometry performed in a sitting position. Participants were allowed at least two tries, and the best one was recorded. FVC, forced expiratory volume in one second (FEV1), and peak expiratory flow (PEF) were measured using an EasyOne® Spirometer (ndd Medical Technologies Inc., MA, USA). Results were converted to predicted values using a validated method [27]. The proportion of FEV1 relative to FVC (FEV%) was calculated to account for subclinical COPD. Respiratory muscle strength was determined by the maximal inspiratory pressure (MIP) and maximal expiratory pressure (MEP), which were measured with the patients in a sitting position using a respiratory pressure meter (MicroRPM™, Care Fusion, Sollentuna, Sweden) [28].

Breathing movements at rest and during maximal breathing were measured using a respiratory movement measuring instrument, RMMI® (ReMo Inc. Keldnaholt, Reykjavik, Iceland) [29]. When using the RMMI, patients with bilateral injuries were excluded so we could compare the injured side to the non-injured side. The ratio of the measure on the injured side to the measure on the non-injured side on the same level was calculated and the values compared between the muscle-sparing surgery and conventional surgery cohorts. The range of motion in the thorax was assessed by measuring thoracic excursion, thoracic flexion, and lateral flexion in a standardized manner [30]. The range of motion in the shoulders was measured with the Boström summary score [31], in which five modalities of movement are graded from 1 to 6, with 6 being the best. Results are reported bilaterally, resulting in a maximum score of 60. Physical activity was assessed by the Grimby activity scale [32], which is a 6-grade scale in which higher numbers indicate a higher level of activity. Physical function was assessed with the Disability Rating Index (DRI) questionnaire [33], which consists of 12 questions, each graded from 0–100 on a visual analog scale (VAS). The score is calculated as a mean of these 12 values, with higher scores representing more disability. Patients also answered a standardized questionnaire concerning pain, local tenderness, breathlessness, pain relief medication, time of return to work, and how much they were able to work. A QoL assessment was carried out with the EQ-5D in both cohorts and converted to an index for analysis. EQ-5D-5L responses were collected for all but the earliest 12 control patients, for whom EQ-5D-3L was used. Cases were converted to a single-summary index using the time trade-off technique with Swedish reference value sets [34, 35]. An EQ-5D VAS was also recorded by the participants [36]. A low radiation dose CT scan of the thorax without intravenous contrast medium was performed after six months in the conventional surgery patients and after 12 months in the muscle-sparing surgery patients. All fractures, both the surgically repaired fractures and the fractures that were not surgically fixated, were assessed for healing. Fractures were defined as healed when no visible fracture line was seen on CT. Fractures with no sign of healing and fractures with signs of partial healing were defined as non-healed.

### Statistical analysis

Data were analyzed using Statistical Package for the Social Sciences (SPSS) version 28.0 (IBM® 2020, Chicago, USA). Results are presented as the mean and standard deviation (SD) for continuous, normally distributed variables and as the median and range for continuous non-parametric variables. Univariate analyses were used to compare continuous variables in a T-test or Mann–Whitney U test. Categorical variables are presented as the number and proportion (%) and compared using Pearson’s chi-squared and Fisher’s exact test. Multiple linear regression and binary logistic regression were used to control for confounding factors. In cases in which the dependent variable was not normally distributed, Blom’s formula was used to normalize the data. *P* < 0.05 was considered significant.

## Results

A total of 50 patients who underwent muscle-sparing surgery for flail chest at our institution during 2020–2022 were eligible for inclusion and enrolled in this study. Nine of these patients opted out of the study before follow-up began. One patient fulfilled one of the exclusion criteria (head AIS > 3) and was excluded from the analysis. Of the remaining 40 patients, all went to both follow-up appointments, except one who did not show up for the second (but was included in the study). We identified 57 patients who underwent conventional surgery for flail chest at our institution from 2010–2017 and were included in previous prospective studies as candidates for the second cohort. Of these, 11 did not fulfill the inclusion criteria (no thoracotomy) and eight fulfilled one of the exclusion criteria (head AIS > 3, *n* = 2; lung resection, *n* = 6). Thus, the conventional surgery cohort had 38 patients.

**Fig. 1:**
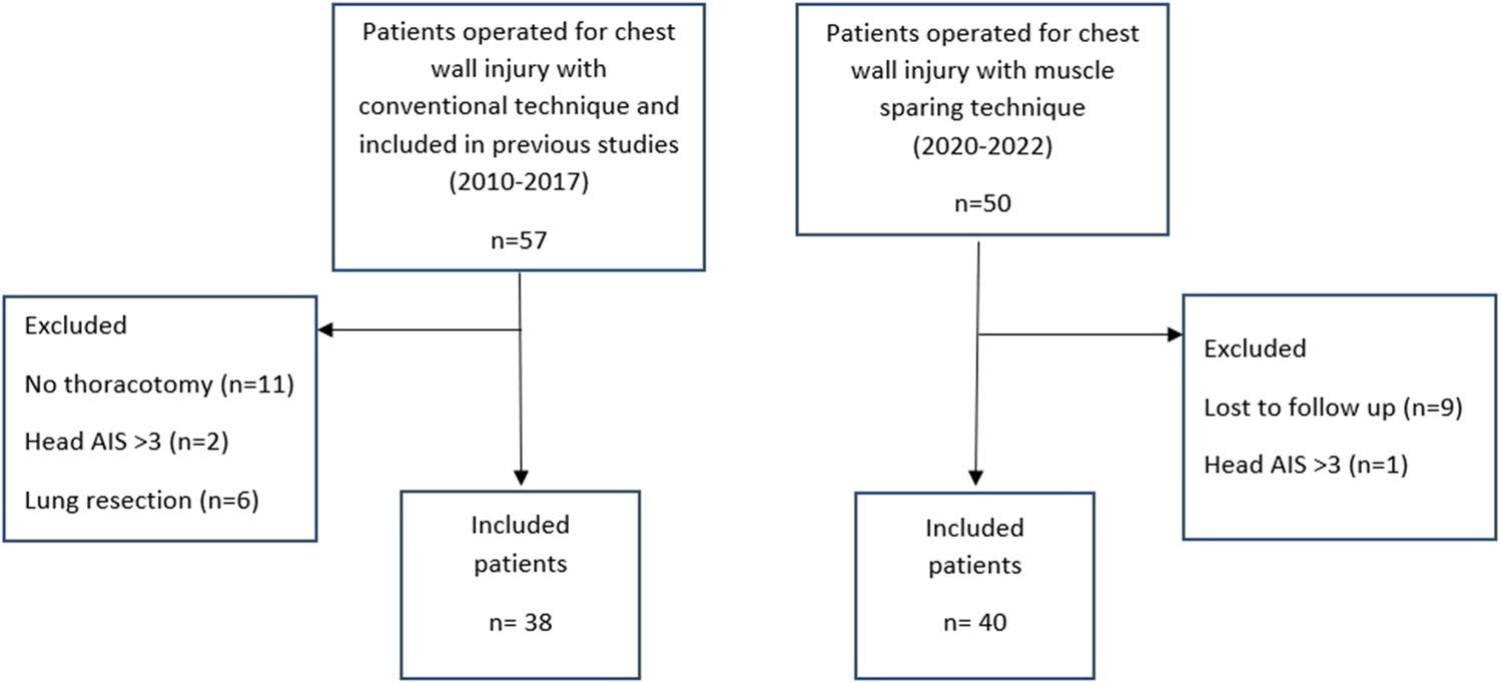
Flowchart of patient inclusion

Our analysis included a total of 78 patients (67.9% men and 32.1% women) with a mean age of 63.6 ± 14.0 years (Fig. 1).

The distribution of sex, age, and comorbidities was similar between the two cohorts, as was the grade of thoracic injuries measured by chest AIS and the number of rib fractures. However, smoking status and injury grade estimated by the ISS and NISS significantly differed between the two surgical groups, as did the day of surgery and the percentage of fractured ribs that were surgically repaired. (Table 1).

**Table 1:** Baseline data for all patients. Comparison between patients operated with a conventional, non-muscle-sparing method with thoracotomy and patients operated with a muscle-sparing method of SSRF

All included patients				
Variables		Conventional (*n* = 38)	Muscle- sparing (*n* = 40)	*p*-value
Sex	Male Female	27 (71.1%) 11 (28.9%)	26 (65.0%) 14 (35.0%)	0.567
Age		62.3 ± 13.7	64.8 ± 14.4	0.444
BMI		25.4 ± 3.6^1^	25.3 ± 3.2	0.979
Smoking	No Yes Previous	14 (37.8%)^1^ 14 (37.8%) 9 (24.3%)	33 (82.5%) 5 (12.5%) 2 (5.0%)	< 0.001*
COPD		2 (5.3%)	3 (7.5%)	1.000^§^
Lung emphysema		0	0	
Asthma		3 (7.9%)	2 (5.0%)	0.671^§^
Diabetes		4 (10.5%)	7 (17.5%)	0.376
ISS		24 (9–50)	17 (9–30)	0.004*
NISS		34 (22–59)	27 (14–59)	0.002*
Head AIS		0 (0–3)	0 (0–3)	0.221
Chest AIS		4 (3–5)	3 (3–5)	0.096
Fractured ribs L		7 (0–12)	6 (0–12)	0.097
Fractured ribs R		4 (0–10)	4.5 (0–11)	0.866
Fractured ribs bilat		12 (31.6%)	11 (27.5%)	0.693
Pneumothorax		27 (71.1%)	31 (77.5%)	0.515
Haemothorax		34 (89.5%)	33 (82.5%)	0.376
Operation day		4 (1–14)	3 (1–7)	0.017*
Ribs operated L		3.5 (0–6)	2 (0–6)	0.007*
Ribs operated R		0 (0–5)	2 (0–5)	0.983
Ribs operated (%)		50 (30–100)	38 (17–100)	0.001*

Baseline data for all included patients. *SSRF* = surgical stabilization of rib fractures, *COPD* = Chronic Obstructive Pulmonary Disease, *BMI* = Body Mass Index, *ISS* = Injury Severity Score, *NISS*, New Injury Severity Score, *AIS* = Abbreviated Injury Score. Categorical variables were analysed with Chi^2^-test or Fisher’s exact test, normally distributed continuous variables with T-test and non-parametric variables with Mann–Whitney U test

^§^Fisher’s exact test

^1^ = 1–3 missing

* = significant valu

After six months, we found no significant difference in predicted FVC, predicted FEV1, or predicted PEF between muscle-sparing and conventional surgery. After 12 months, patients who underwent muscle-sparing surgery had a significantly higher predicted FVC than those who underwent conventional surgery (92.3 ± 14.3 vs. 85.0 ± 15.3, *p* = 0.037). We did not find significant differences between the other spirometry variables (Table 2). Multiple linear regression analysis showed that surgical technique made a significant contribution to the difference in predicted FVC at 12 months when controlling for the confounding factors age, sex, NISS, smoking status, and days from trauma to surgery (*p* = 0.042). These factors were chosen because they can be assumed to influence outcomes according to previous research and/or were markedly different between the two cohorts [19, 26, 37, 38]. Preliminary analyses were conducted to ensure the assumptions of normality, linearity, multicollinearity, and homoscedasticity were not violated.

**Table 2:** Comparison of lung function between patients operated with a conventional, non-muscle-sparing method with thoracotomy and patients operated with a muscle-sparing method of SSRF. Multiple linear regression performed to assess whether operation technique significantly explains a change in outcome while controlling for age, sex, smoking status, NISS and number of days from trauma to surgery

Variable		Conventional (n 6 m = 37, 12 m = 36)	Muscle-sparing (n 6 m = 40, 12 m = 39)	*p*-value	Adjusted p-value
FVC% pred	**6 months** **12 months**	84.5 ± 17.5 85.0 ± 15.3	90.8 ± 16.7 92.3 ± 14.3	0.108 0.037*	0.185 0.042*
FEV1% pred	**6 months** **12 months**	78.4 ± 18.9 79.7 ± 16.2	83.4 ± 17.3 86.0 ± 16.5	0.223 0.100	0.370 0.081
PEF% pred	**6 months** **12 months**	86.1 ± 23.2 94.1 ± 17.5	89.7 ± 26.0 97.0 ± 25.6^1^	0.531 0.571	0.325 0.877

Lung function measured with spirometry. *SSRF* = surgical stabilization of rib fractures, *NISS* = New Injury Severity Score *FVC* = Forced Vital Capacity, pred = predicted, *FEV1* = Forced Expiratory Volume in 1 s, *PEF* = Peak Expiratory Flow. Normally distributed continuous variables were analysed with t-test. Standard multiple linear regression was performed

^1^ = 1 missing

* = significant value

The FEV% at 12 months was analyzed and no significant difference found between the muscle-sparing surgery cohort and the conventional surgery cohort (0.74 ± 0.1 vs. 0.74 ± 0.1, *p* = 0.859). When the multiple linear regression was adjusted for age, sex, NISS, smoking status, and days from trauma to surgery, the difference was not significant (*p* = 0.909).

Regarding the secondary outcomes, shoulder mobility measured with the Boström index was significantly better in the muscle-sparing surgery cohort than in the conventional surgery cohort at 6 months [58 (30–60) vs. 55 (37–60); p = 0.006]. A significant difference was still present at 12 months [59 (41–60) vs. 56 (42–60), respectively; *p* = 0.007]. Multiple linear regression was performed to assess whether surgical technique still made a significant contribution to the variance in the Boström index while controlling for the confounders age, sex, NISS, smoking status, and days from trauma to surgery. The adjusted analysis showed that surgical technique had a significant impact on the Boström index at 12 months (*p* = 0.015), but not at six months (*p* = 0.071; Table 3).

**Table 3:** Comparison of shoulder and thoracic mobility, respiratory muscle strength and presence of winged scapula between patients operated with a conventional, non-muscle-sparing method with thoracotomy and patients operated with a muscle-sparing method of SSRF. Adjusted for age, sex, smoking status, NISS and days from trauma to surgery

Variable		Conventional (*n* = 38)	*N* =	Muscle-sparing (*n* = 40)	*N* =	*p*-value	Adjusted *p*-value
Boström index	**6 m** **12 m**	55 (37–60) 56 (36–60)	29 35	58 (30–60) 59 (41–60)	40 39	0.006* 0.007*	0.071 0.015*
Upper thoracic excursion	**6 m** **12 m**	3.2 ± 1.4 3.1 ± 1.4	37 36	3.2 ± 1.7 3.3 ± 1.8	40 39	0.933 0.481	0.651 0.852
Lower thoracic excursion	**6 m** **12 m**	4.1 ± 2.2 3.8 ± 2.0	37 35	4.2 ± 2.2 4.5 ± 2.2	40 39	0.771 0.140	0.254 0.082
Thoracic flexion	**6 m** **12 m**	1.9 ± 0.8 1.9 ± 0.8	36 36	1.7 ± 0.8 1.7 ± 1.0	40 39	0.197 0.311	0.524 0.199
Thoracic extension	**6 m** **12 m**	1.1 ± 0.8 1.1 ± 0.6	37 36	1.5 ± 1.1 1.1 ± 0.7	40 39	0.109 0.980	0.289 0.803
Lateral flexion injured side	**6 m** **12 m**	12.6 ± 4.2 11.4 ± 5.6	26 24	15.9 ± 4.9 16.1 ± 5.4	28 28	0.010* 0.004*	0.041* 0.010*
Lateral flexion non-injured side	**6 m** **12 m**	12.7 ± 5.2 11.7 ± 5.1	26 24	16.5 ± 5.0 15.8 ± 4.9	28 28	0.009* 0.006*	0.024* 0.002*
MIP	**6 m** **12 m**	73.3 ± 38.5 79.3 ± 29.3	28 27	79.2 ± 30.3 81.2 ± 29.7	40 39	0.477 0.806	0.231 0.247
MEP	**6 m** **12 m**	104.1 ± 39.6 108.1 ± 39.8	28 27	111.9 ± 37.7 116.3 ± 42.6	40 39	0.414 0.438	0.385 0.203
Winged scapula injured side	**6 m** **12 m**	1 (3.6%) 2 (7.1%)	28 28	1 (2.5%) 0	40 39	1.000^§^	0.423
Winged scapula non-injured side	**6 m** **12 m**	1 (3.6%) 1 (3.6%)	28 28	0 0	40 39		

Measurements of mobility, respiratory strength, occurrence of winged scapula and wound healing. Bilateral injuries excluded from analysis of lateral flexion. *SSRF* = surgical stabilization of rib fractures, *MIP* = Maximum Inspiratory Pressure, *MEP* = Maximum Expiratory Pressure, *CT* = Computed tomography. Categorical variables were analysed with Chi^2^-test or Fisher’s exact test, normally distributed continuous variables with T-test and non-parametric variables with Mann–Whitney U test. Multiple linear regression was performed on normally distributed, continuous variables. Continuous variables with non-normal distribution were normalized using Blom’s formula prior to regression analysis. Binary logistic regression was performed on categorical variables

^§^Fisher’s exact test

* = significant value

The patients who underwent muscle-sparing surgery had greater lateral thoracic flexion on the injured side after six months compared with those who underwent conventional surgery (15.9 ± 4.9 cm vs. 12.6 ± 4.2 cm, *p* = 0.010). The difference was still significant (*p* = 0.003) after multiple linear regression adjusted for age, sex, NISS, smoking status, and days from trauma to surgery (Table 3). The patients who underwent muscle-sparing surgery also had greater lateral flexion on the non-injured side (15.7 ± 4.9 cm vs. 12.5 ± 3.7 cm with conventional surgery, *p* = 0.009). Multiple linear regression was performed to adjust for age, sex, NISS, smoking status and days from trauma to surgery, and the difference was still significant (*p* = 0.024). These differences all persisted at 12 months, with the adjusted difference still significant on the injured side (*p* < 0.001) and non-injured side (*p* = 0.002, Table 3).

We found no significant differences between the surgical groups with regards to respiratory muscle strength, the presence of winged scapula (Table 3), or breathing movements (Table 4) at six or 12 months. In 22 of the patients in the muscle-sparing surgery cohort (81.5%), all fractures were healed on the CT scan at 1 year. In the conventional surgery cohort, all fractures were healed on the CT scan in 12 (57.1%) patients at 6 months.

**Table 4:** Comparison of breathing movements, ratio of the measure on the injured side to the measure on the non-injured side for patients operated with a non-muscle-sparing method with thoracotomy versus patients operated with a muscle-sparing method of SSRF. Adjusted for age, sex, smoking status, NISS and days from trauma to surgery

**Breathing movements at rest**	**Op technique**	**6 m**	***p*-value**	**Adjusted** ***p*-value**	**12 m**	***p*-value**	**Adjusted** ***p*-value**
Upper thorax (%)	**Muscle-sparing**	87.8 ± 25.5	0.418	0.937	95.9 ± 27.2	0.757	0.443
	**Conventional**	94.8 ± 37.6			93.3 ± 32.0		
Lower thorax (%)	**Muscle-sparing**	95.8 ± 43.0	0.342	0.715	104.0 ± 64.9	0.289	0.692
	**Conventional**	86.5 ± 26.1			89.9 ± 23.1		
Abdomen (%)	**Muscle-sparing**	96.4 ± 17.5	0.662	0.687	95.8 ± 14.0	0.271	0.785
	**Conventional**	99.1 ± 27.0			105.8 ± 41.5		
**Breathing movements at max**	**Op technique**	**6 m**	***p*-value**	**Adjusted *p*-value**	**12 m**	***p*-value**	**Adjusted *p*-value**
Upper thorax (%)	**Muscle-sparing**	108.3 ± 100.1	0.247	0.443	90.7 ± 21.5	0.579	0.126
	**Conventional**	85.0 ± 14.7			94.1 ± 22.1		
Lower thorax (%)	**Muscle-sparing**	93.8 ± 31.2	0.830	0.586	90.1 ± 36.5^1^	0.544	0.921
	**Conventional**	95.6 ± 26.9			96.9 ± 41.9		
Abdomen (%)	**Muscle-sparing**	99.4 ± 27.0	0.582	0.588	94.4 ± 18.6	0.453	0.752
	**Conventional**	95.7 ± 16.4			98.9 ± 24.2		

Analysis of breathing movements. Values were analysed as percentage of movement on injured side vs non-injured side. All bilateral injuries were excluded for this analysis. Normally distributed continuous variables were analysed with t-test. *SSRF* = surgical stabilization of rib fractures. Multiple linear regression was performed on normally distributed, continuous variables

n conventional 6 m = 26

n muscle sparing 6 m = 29

n conventional 12 m = 24

n muscle sparing 12 m = 28

^1^ = 1 missing

* = significant value

Regarding patient-reported outcomes, we did not find significant differences in physical function, physical activity, pain at rest, pain while breathing, breathlessness, or use of pain relief medication at six or 12 months (Table 5). The patients who underwent muscle-sparing surgery had significantly better EQ-5D and EQ-5D VAS scores at six months. We did not find a significant difference in the EQ-5D (*p* = 0.074) or EQ-5D VAS (*p* = 0.237) scores after adjusting for age, sex, NISS, smoking status, and days from trauma to surgery in multiple linear regression (Table 5). At 12 months, we found no significant differences in the EQ-5D or EQ-5D VAS scores.

**Table 5:** Comparison of physical function, physical activity, quality of life, pain and breathlessness between patients operated with a non-muscle-sparing method with thoracotomy and patients operated with a muscle-sparing method of SSRF

Variable		N	Conventional	N	Muscle-sparing	*p*-value	Adjusted *p*-value
DRI	**6 m** **12 m**	36 36	26.5 (0–65.3) 21.4 (0–62.0)	40 39	20.8 (0–86.3) 16.7 (0–70.4)	0.223 0.856	0.438 0.891
Grimby	**6 m** **12 m**	37 36	3.8 ± 0.9 4.3 ± 1.2	40 39	4.1 ± 1.2 4.1 ± 1.0	0.300 0.559	0.520 0.305
EQ5D index	**6 m** **12 m**	36 36	0.89 (0.52–0.98)^1^ 0.91 (0.66–0.98)^1^	40 39	0.94 (0.39–0.98) 0.93 (0.38–0.98)	0.018* 0.242	0.074 0.733
EQ5D VAS	**6 m** **12 m**	36 36	75 (20–90) 80.0 (30–100)	40 39	81.0 (20–100) 81.0 (20–100)	0.030* 0.351	0.237 0.622
Pain at rest	**6 m** **12 m**	29 29	2 (6.9%) 2 (6.9%)	40 39	3 (7.5%) 5 (12.8%)	1.000^§^ 0.690^§^	0.743 0.665
Pain while breathing	**6 m** **12 m**	29 29	3 (10.3%) 3 (10.3%)	40 39	4 (10.0%) 3 (7.7%)	1.000^§^ 1.000^§^	0.545 0.868
Local tenderness	**6 m** **12 m**	29 29	16 (55.2%) 11 (37.9%)	40 39	12 (30.0%) 11 (28.2%)	0.036* 0.397	0.530 0.923
Breathlessness	**6 m** **12 m**	29 29	8 (27.6%) 5 (17.2%)	40 39	11(27.5%) 8 (20.5%)	0.994 0.734	0.517 0.731
Pain medication	**6 m** **12 m**	29 29	4 (13.8%) 3 (10.3%)	40 38	10 (25.0%) 8 (21.1%)	0.253 0.326^§^	0.252 0.680

Analysis of patient reported outcomes. *SSRF* = surgical stabilization of rib fractures, *DRI* = Disability Rating Index, *Grimby* = Grimby activity scale, *VAS* = Visual Analog Scale. Categorical variables were analysed with Chi^2^-test or Fisher’s exact test, normally distributed continuous variables with T-test and non-parametric variables with Mann–Whitney U test. Multiple linear regression was performed on normally distributed, continuous variables. Continuous variables with non-normal distribution were normalized using Blom’s formula prior to regression analysis. Binary logistic regression was performed on categorical variables

^§=^Fisher’s exact test

* = significant value

^1^ = n = 8 evaluated with EQ-5D-3L

The patients who underwent conventional surgery had a significantly higher incidence of local tenderness at six months (Table 5). Binary logistic regression was used to control for age, sex, NISS, smoking status, and days from trauma to surgery. The adjusted difference was not significant (*p* = 0.530). No difference was present at 12 months.

We did not find a significant difference between the surgical groups regarding return to work, with the muscle-sparing surgery patients returning to work after a mean 64.1 ± 54.7 days (*n* = 12) and the conventional surgery patients after a mean 84.4 ± 47.8 days (*n* = 12; *p* = 0.343). In the muscle-sparing surgery cohort, patients worked a median of 100% (range 70–100, *n* = 12) after six months, and in the conventional surgery cohort, patients worked a median of 100% (range 0–100, *n* = 14) after six months (*p* = 0.494). Patients not working before the chest trauma were excluded from both analyses.

## Discussion

In this cohort study of prospectively included patients with historical controls, we compared two surgical methods for stabilization of flail chest and found that the muscle-sparing method was associated with better long-term lung function, shoulder mobility, and thoracic mobility. An abundance of studies have described different methods for rib plating [11, 13, 16, 39, 40], but studies comparing surgical techniques are lacking. Less invasive surgical techniques are advantageous in rib fixation with regards to hospital LOS, ICU LOS, and the time on a ventilator. Less invasiveness is also associated with shorter surgical times and less pain compared to an open approach [19–21]. Some techniques use a thoracoscopic approach [20, 21], but a muscle-sparing open method has also been described [19]. However, studies comparing long-term outcomes are scarce. The present study indicates that surgical technique matters, and that a muscle-sparing surgical procedure has long-term advantages.

Patients who underwent surgery with the muscle-sparing method had better predicted FVC than patients who underwent surgery based on the conventional method with thoracotomy. Previous randomized trials have found that surgery is associated with better postoperative lung function compared to nonoperative management [1, 2]. However, not all chest wall surgeries are equal. Thoracotomy causes significant morbidity regardless of the method used [41], and this may explain much of the difference in lung function between our study cohorts. Division of the large muscles of the chest wall impairs respiratory function [42]; therefore, choosing the right technique could be crucial.

The conventional surgery cohort was prospectively evaluated at an earlier time than the muscle-sparing surgery cohort, and the two surgical groups differed in several baseline characteristics. We chose to control for potential confounders with standard multiple linear regression and binary logistic regression. The variables age, sex, NISS, smoking status, and days between trauma and surgery were chosen because they were deemed to be important factors with a potentially large impact on outcome. The distribution of pulmonary disease in the muscle-sparing cohort and the conventional cohort was similar (Table 1). A skewed distribution of undiagnosed COPD between the cohorts may affect lung function [38]; however, the FEV% did not differ significantly between the cohorts, so a difference in undiagnosed COPD is unlikely.

Regarding the secondary endpoints, the muscle-sparing method offered an advantage regarding shoulder mobility and thoracic mobility. This is probably due to morbidity from the large, muscle-dividing incisions used in the conventional method. There is some evidence that muscle-sparing thoracotomy causes fewer mobility issues than conventional, posterolateral thoracotomy [41, 43]. More healed rib fractures were seen on CT scans at follow-up in the muscle-sparing surgery patients than the conventional surgery patients. However, the conventional surgery patients underwent the follow-up CT at six months and the muscle-sparing surgery patients had it performed at 12 months, so no real conclusion can be drawn from the discrepancy in fracture healing. Interestingly, the patients who underwent muscle-sparing surgery had fewer fractures repaired but better lung function. This may imply that selection of which rib fractures to stabilize is more important than fixation of as many as possible.

Interestingly, the cohorts did not differ in patient-reported outcomes. This probably indicates that, even though there are measurable differences in outcome between the surgical methods, both are similarly well tolerated in the long term. The EQ5D may be too broad to capture subtle quality of life nuances in these patients. A survey tailored to this specific group could be a valuable future research topic. The muscle-sparing surgery patients returned to work earlier than the conventional surgery patients, but not significantly earlier. This is likely due to the small sample size. Many patients did not work before the trauma event and were excluded from the analysis. Further studies need to be performed to achieve conclusive results.

A strength of this study is its design in which participants were included prospectively and consecutively. This is not a randomized study, and the control cohort consisted of historic patients whose data were collected prospectively. All patients were operated on by experienced surgeons at a high-volume center. Follow-up was conducted in a standardized manner by specialist physiotherapists and specialist surgeons. The study included enough patients to achieve an adequate power according to our calculations.

This study has some limitations. There are reasons to believe that muscle-sparing surgery has a positive outcome on LOS, ventilator treatment, and pneumonia [19]; thus, we deemed it unethical to conduct a randomized, prospective study including the conventional method. We do believe a prospective study with historical controls of this kind can still produce valid results, especially considering the lack of studies on the subject. We performed multiple linear regression to adjust for important confounders. Notably, we did not adjust for the difference in number of ribs fixated. The conventional surgical approach provides wider access to the chest wall and allows for the fixation of more fractures. This is part of the surgical method and should not be considered a confounding factor. We did not register the length of the incisions or their placement, and we did not compare the locations and displacement of fractures. CT examinations were conducted at six months in the conventional surgery patients and 12 months in the muscle-sparing surgery patients, which makes comparative analysis challenging. QoL was assessed by two different methods. Although our national references were used for both, index values for EQ-5D-3L and EQ-5D-5L may differ, which could affect comparisons between the cohorts. However, the EQ VAS scales are completely comparable and not dependent on method.

Many different surgical methods have been described for the repair of chest wall injuries [7–9, 11, 13, 39]. Surgery has been established to lead to better outcomes for properly selected patients [1–3], but there is no clear consensus on the best method. Muscle-sparing surgery seems to be superior to a conventional, non-muscle-sparing method with thoracotomy, with less time spent in the hospital, in the ICU, and on a ventilator [19]. The results from the present study show that a muscle-sparing surgical method is also favorable in the long term and associated with better lung function and greater mobility. The principle of"do no further harm"is crucial in surgery, and this study supports its importance in chest wall surgery. Other areas requiring further evaluation include the role of thoracoscopy, which may be the future of minimally invasive chest wall surgery or a powerful adjunct. Ultrasound imaging is interesting because it permits the localization of fractures in a bedside setting, though whether preoperative marking of fractures or other uses of ultrasound have a place in the care of chest wall injuries is still uncertain.

## Conclusion

Patients who underwent muscle-sparing surgery for traumatic chest wall injuries have better long-term lung function, shoulder mobility, and thoracic mobility than patients who underwent conventional surgery, but not with respect to self-reported outcomes. This indicates that the surgical technique matters for long-term results. We suggest considering a muscle-sparing surgical method when feasible.

## Supplementary Information

Below is the link to the electronic supplementary material.

Supplementary file1 (DOCX 156 KB)

## Data Availability

The data that support the findings of this study are not openly available due to reasons of sensitivity and are available from the corresponding author upon reasonable request. Data are located in controlled access data storage at Sahlgrenska Universitetssjukhuset.
