# Supplementary material for: Comparison of long-term outcome between muscle sparing and non-muscle sparing surgical techniques in rib plating
Source: Eur J Trauma Emerg Surg. 2025 May 20;51(1):212. doi: 10.1007/s00068-025-02881-z (PMC12092516; doi:10.1007/s00068-025-02881-z)
Supplement: Supplementary file 1 — Supplementary file1 (DOCX 156 KB) [file 68_2025_2881_MOESM1_ESM.docx]

Supplementary table 1 - Multiple linear regression assessing whether operative technique significantly explains a difference in forced vital capacity at 6 months while controlling for age, sex, smoking status, NISS and number of days from trauma to surgery.

| Variable | B | SE B | Standardized  Beta | p-value |
| --- | --- | --- | --- | --- |
| Constant  Age  Sex  Smoking  NISS  Op day  Op technique | 105.16  -0.41  12.40  -3.67  0.15  -0.18  4.84 | 10.93  0.14  4.29  2.87  0.18  0.74  4.45 | -0.33  0.34  -0.16  0.09  -0.03  0.14 | <0.001*  0.006*  0.005*  0.204  0.408  0.806  0.281 |

R^2^=0.23, *F* (6, 75) = 3.347, *p* =0.006

Standard multiple linear regression. Dependent variable is predicted forced vital capacity at 6 months. NISS=New Injury Severity Score, Op day=days from trauma to surgery, Op technique=operation technique.

*=significant value

Supplementary table 2 - Multiple linear regression assessing whether operative technique significantly explains a difference in forced vital capacity at 12 months while controlling for age, sex, smoking status, NISS and number of days from trauma to surgery.

| Variable | B | SE B | Standardized  Beta | p-value |
| --- | --- | --- | --- | --- |
| Constant  Age  Sex  Smoking  NISS  Op day  Op technique | 107.23  -0.52  9.71  -3.89  0.29  0.14  7.50 | 8.88  0.12  3.49  2.33  0.15  0.60  3.62 | -0.48  0.30  -0.19  0.20  0.03  0.25 | <0.001*  <0.001*  0.007*  0.100  0.060  0.814  0.042* |

R^2^=0.35, *F* (6, 73) = 6.085, *p* <0.001

Standard multiple linear regression. Dependent variable is predicted forced vital capacity at 12 months. NISS=New Injury Severity Score, Op day=days from trauma to surgery, Op technique=operation technique.

*=significant value

Supplementary table 3 - Multiple linear regression assessing whether operative technique significantly explains a difference in predicted forced expiratory volume in one second at 6 months while controlling for age, sex, smoking status, NISS and number of days from trauma to surgery.

| Variable | B | SE B | Standardized  Beta | p-value |
| --- | --- | --- | --- | --- |
| Constant  Age  Sex  Smoking  NISS  Op day  Op technique | 100.48  -0.46  6.53  -5.88  0.20  0.51  4.29 | 11.68  0.15  4.59  3.06  0.20  0.79  4.76 | -0.36  0.17  -0.24  0.12  0.08  0.12 | <0.001*  0.004*  0.159  0.059  0.301  0.526  0.370 |

R^2^=0.20, *F* (6, 75) = 2.905, *p* =0.014

Standard multiple linear regression. Dependent variable is predicted forced expiratory volume in one second at 6 months. NISS=New Injury Severity Score, Op day=days from trauma to surgery, Op technique=operation technique.

*=significant value

Supplementary table 4 - Multiple linear regression assessing whether operative technique significantly explains a difference in predicted forced expiratory volume in one second at 12 months while controlling for age, sex, smoking status, NISS and number of days from trauma to surgery.

R^2^=0.28, *F* (6, 73) = 4.348, *p* <0.001

| Variable | B | SE B | Standardized  Beta | p-value |
| --- | --- | --- | --- | --- |
| Constant  Age  Sex  Smoking  NISS  Op day  Op technique | 102.85  -0.53  2.94  -5.03  0.27  0.80  7.41 | 10.27  0.14  4.04  2.69  0.17  0.70  4.18 | -0.45  0.08  -0.22  0.17  0.13  0.23 | <0.001*  <0.001*  0.468  0.066  0.128  0.255  0.081 |

Standard multiple linear regression. Dependent variable is predicted forced expiratory volume in one second at 12 months. NISS=New Injury Severity Score, Op day=days from trauma to surgery, Op technique=operation technique.

*=significant value

Supplementary table 5 - Multiple linear regression assessing whether operative technique significantly explains a difference in predicted peak expiratory flow at 6 months while controlling for age, sex, smoking status, NISS and number of days from trauma to surgery.

| Variable | B | SE B | Standardized  Beta | p-value |
| --- | --- | --- | --- | --- |
| Constant  Age  Sex  Smoking  NISS  Op day  Op technique | 126.25  -0.78  -0.14  -0.50  0.34  -0.53  6.26 | 15.50  0.20  6.09  4.06  0.26  1.05  6.32 | -0.45  -0.00  -0.02  0.15  -0.06  0.13 | <0.001*  <0.001*  0.982  0.903  0.193  0.619  0.325 |

R^2^=0.23, *F* (6, 75) = 3.431, *p* =0.005

Standard multiple linear regression. Dependent variable is predicted peak expiratory flow at 6 months. NISS=New Injury Severity Score, Op day=days from trauma to surgery, Op technique=operation technique.

*=significant value

Supplementary table 6 - Multiple linear regression assessing whether operative technique significantly explains a difference in predicted peak expiratory flow at 12 months while controlling for age, sex, smoking status, NISS and number of days from trauma to surgery.

| Variable | B | SE B | Standardized  Beta | p-value |
| --- | --- | --- | --- | --- |
| Constant  Age  Sex  Smoking  NISS  Op day  Op technique | 113.96  -0.38  -4.27  -7.86  0.39  -0.29  0.92 | 14.55  0.19  5.72  3.81  0.24  0.99  5.93 | -0.25  -0.09  -0.26  0.19  -0.04  0.02 | <0.001*  0.049*  0.458  0.043*  0.116  0.769  0.877 |

R^2^=0.18, *F* (6, 72) = 2.478, *p* =0.032

Standard multiple linear regression. Dependent variable is predicted peak expiratory flow at 12 months. NISS=New Injury Severity Score, Op day=days from trauma to surgery, Op technique=operation technique.

*=significant value

| Variable | B | SE B | Standardized  Beta | p-value |
| --- | --- | --- | --- | --- |
| Constant  Age  Sex  Smoking  NISS  Op day  Op technique | 1.583  -0.02  0.03  -0.22  -0.01  -0.02  0.46 | 0.618  0.01  0.24  0.16  0.01  0.04  0.25 | -0.33  -0.01  -0.17  -0.09  -0.50  0.25 | 0.013*  0.008*  0.912  0.186  0.466  0.630  0.071 |

Supplementary table 7 - Multiple linear regression assessing whether operative technique significantly explains a difference in Boström index at six months while controlling for age, sex, smoking status, NISS and number of days from trauma to surgery.

R^2^=0.27, *F* (6, 67) = 3.824, *p* =0.003

Standard multiple linear regression. Dependent variable is Boström index at six months. Non-normal data was normalized using Blom’s formula prior to regression analysis. NISS=New Injury Severity Score, Op day=days from trauma to surgery, Op technique=operation technique.

*=significant value

| Variable | B | SE B | Standardized  Beta | p-value |
| --- | --- | --- | --- | --- |
| Constant  Age  Sex  Smoking  NISS  Op day  Op technique | 1.21  -0.02  0.23  0.01  -0.00  -0.01  0.60 | 0.59  0.01  0.23  0.15  0.01  0.04  0.24 | -0.37  0.12  0.01  -0.03  -0.01  0.33 | 0.045*  0.003*  0.324  0.926  0.823  0.910  0.015* |

Supplementary table 8 - Multiple linear regression assessing whether operative technique significantly explains a difference in Boström index at 12 months while controlling for age, sex, smoking status, NISS and number of days from trauma to surgery.

R^2^=0.22, *F* (6, 72) = 3.135, *p* =0.009

Standard multiple linear regression. Dependent variable is Boström index at 12 months. Non-normal data was normalized using Blom’s formula prior to regression analysis. NISS=New Injury Severity Score, Op day=days from trauma to surgery, Op technique=operation technique.

*=significant value

| Variable | B | SE B | Standardized  Beta | p-value |
| --- | --- | --- | --- | --- |
| Constant  Age  Sex  Smoking  NISS  Op day  Op technique | 6.01  -0.04  0.05  -0.46  0.00  0.01  -0.19 | 1.00  0.01  0.39  0.26  0.02  0.07  0.41 | -0.37  0.01  -0.22  0.02  0.02  -0.06 | <0.001*  0.003*  0.908  0.087  0.850  0.891  0.651 |

Supplementary table 9 - Multiple linear regression assessing whether operative technique significantly explains a difference in upper thoracic excursion at six months while controlling for age, sex, smoking status, NISS and number of days from trauma to surgery.

R^2^=0.19, *F* (6, 75) = 2.605, *p* =0.025

Standard multiple linear regression. Dependent variable is upper thoracic excursion at six months. NISS=New Injury Severity Score, Op day=days from trauma to surgery, Op technique=operation technique.

*=significant value

| Variable | B | SE B | Standardized  Beta | p-value |
| --- | --- | --- | --- | --- |
| Constant  Age  Sex  Smoking  NISS  Op day  Op technique | 6.56  -0.04  -0.46  -0.24  -0.01  -0.05  0.08 | 1.06  0.01  0.42  0.28  0.02  0.07  0.43 | -0.35  -0.13  -0.11  -0.07  -0.08  0.03 | <0.001*  0.005*  0.268  0.389  0.536  0.509  0.852 |

Supplementary table 10 - Multiple linear regression assessing whether operative technique significantly explains a difference in upper thoracic excursion at 12 months while controlling for age, sex, smoking status, NISS and number of days from trauma to surgery.

R^2^=0.22, *F* (6, 73) = 3.098, *p* =0.010

Standard multiple linear regression. Dependent variable is upper thoracic excursion at 12 months. NISS=New Injury Severity Score, Op day=days from trauma to surgery, Op technique=operation technique.

*=significant value

| Variable | B | SE B | Standardized  Beta | p-value |
| --- | --- | --- | --- | --- |
| Constant  Age  Sex  Smoking  NISS  Op day  Op technique | 6.07  -0.06  -0.66  -0.49  0.04  0.19  0.62 | 1.33  0.02  0.52  0.35  0.02  0.09  0.54 | -0.39  -0.14  -0.17  0.20  0.24  0.15 | <0.001*  <0.001*  0.213  0.164  0.079  0.044*  0.254 |

Supplementary table 11 - Multiple linear regression assessing whether operative technique significantly explains a difference in lower thoracic excursion at six months while controlling for age, sex, smoking status, NISS and number of days from trauma to surgery.

R^2^=0.25, *F* (6, 75) = 3.912, *p* =0.002

Standard multiple linear regression. Dependent variable is lower thoracic excursion at six months. NISS=New Injury Severity Score, Op day=days from trauma to surgery, Op technique=operation technique.

*=significant value

| Variable | B | SE B | Standardized  Beta | p-value |
| --- | --- | --- | --- | --- |
| Constant  Age  Sex  Smoking  NISS  Op day  Op technique | 6.67  -0.05  -1.18  -0.13  0.01  0.03  0.95 | 1.32  0.02  0.52  0.35  0.02  0.09  0.54 | -0.32  -0.27  -0.05  0.06  0.04  0.23 | <0.001*  0.007*  0.027*  0.715  0.610  0.755  0.082 |

Supplementary table 12 - Multiple linear regression assessing whether operative technique significantly explains a difference in lower thoracic excursion at 12 months while controlling for age, sex, smoking status, NISS and number of days from trauma to surgery.

R^2^=0.26, *F* (6, 72) = 3.818, *p* =0.003

Standard multiple linear regression. Dependent variable is lower thoracic excursion at 12 months. NISS=New Injury Severity Score, Op day=days from trauma to surgery, Op technique=operation technique.

*=significant value

| Variable | B | SE B | Standardized  Beta | p-value |
| --- | --- | --- | --- | --- |
| Constant  Age  Sex  Smoking  NISS  Op day  Op technique | 2.62  -0.01  0.26  0.05  -0.00  0.04  -0.15 | 0.57  0.01  0.22  0.15  0.01  0.04  0.23 | -0.24  -0.15  -0.04  -0.05  0.12  -0.09 | <0.001*  0.066  0.241  0.743  0.691  0.347  0.524 |

Supplementary table 13 - Multiple linear regression assessing whether operative technique significantly explains a difference in thoracic flexion at six months while controlling for age, sex, smoking status, NISS and number of days from trauma to surgery.

R^2^=0.09, *F* (6, 74) = 1.110, *p* =0.366

Standard multiple linear regression. Dependent variable is thoracic flexion at six months. NISS=New Injury Severity Score, Op day=days from trauma to surgery, Op technique=operation technique.

*=significant value

| Variable | B | SE B | Standardized  Beta | p-value |
| --- | --- | --- | --- | --- |
| Constant  Age  Sex  Smoking  NISS  Op day  Op technique | 2.65  -0.01  -0.07  -0.30  -0.01  0.05  -0.35 | 0.66  0.01  0.26  0.17  0.01  0.04  0.27 | -0.10  -0.03  -0.23  -0.09  0.15  -0.19 | <0.001*  0.430  0.789  0.086  0.457  0.261  0.199 |

Supplementary table 14 - Multiple linear regression assessing whether operative technique significantly explains a difference in thoracic flexion at 12 months while controlling for age, sex, smoking status, NISS and number of days from trauma to surgery.

R^2^=0.09, *F* (6, 73) = 1.121, *p* =0.360

Standard multiple linear regression. Dependent variable is thoracic flexion at 12 months. NISS=New Injury Severity Score, Op day=days from trauma to surgery, Op technique=operation technique.

*=significant value

| Variable | B | SE B | Standardized  Beta | p-value |
| --- | --- | --- | --- | --- |
| Constant  Age  Sex  Smoking  NISS  Op day  Op technique | 2.46  -0.01  0.35  0.00  -0.02  0.02  0.28 | 0.65  0.01  0.26  0.17  0.01  0.04  0.27 | -0.20  0.17  0.00  -0.20  0.05  0.15 | <0.001*  0.105  0.176  0.983  0.092  0.724  0.289 |

Supplementary table 15 - Multiple linear regression assessing whether operative technique significantly explains a difference in thoracic extension at six months while controlling for age, sex, smoking status, NISS and number of days from trauma to surgery.

R^2^=0.13, *F* (6, 75) = 1.646, *p* =0.148

Standard multiple linear regression. Dependent variable is thoracic extension at six months. NISS=New Injury Severity Score, Op day=days from trauma to surgery, Op technique=operation technique.

*=significant value

| Variable | B | SE B | Standardized  Beta | p-value |
| --- | --- | --- | --- | --- |
| Constant  Age  Sex  Smoking  NISS  Op day  Op technique | 2.45  -0.02  0.10  -0.07  -0.01  0.01  -0.04 | 0.43  0.01  0.17  0.11  0.01  0.03  0.18 | -0.38  0.07  -0.08  -0.16  0.05  -0.03 | <0.001*  0.003*  0.549  0.543  0.179  0.706  0.803 |

Supplementary table 16 - Multiple linear regression assessing whether operative technique significantly explains a difference in thoracic extension at 12 months while controlling for age, sex, smoking status, NISS and number of days from trauma to surgery.

R^2^=0.17, *F* (6, 73) = 2.241, *p* =0.050

Standard multiple linear regression. Dependent variable is thoracic extension at 12 months. NISS=New Injury Severity Score, Op day=days from trauma to surgery, Op technique=operation technique.

*=significant value

Supplementary table 17 - Multiple linear regression assessing whether operative technique significantly explains a difference in lateral flexion on the injured side at six months while controlling for age, sex, smoking status, NISS and number of days from trauma to surgery.

R^2^=0.38, *F* (6, 53) = 4.786, *p* <0.001

| Variable | B | SE B | Standardized  Beta | p-value |
| --- | --- | --- | --- | --- |
| Constant  Age  Sex  Smoking  NISS  Op day  Op technique | 25.12  -0.17  1.00  0.52  -0.06  -0.06  2.83 | 3.26  0.05  1.59  0.89  0.07  0.21  1.35 | -0.52  0.09  0.08  -0.12  0.04  0.29 | <0.001*  <0.001*  0.531  0.560  0.333  0.780  0.041* |

Standard multiple linear regression. Dependent variable is lateral flexion on the injured side at six months. NISS=New Injury Severity Score, Op day=days from trauma to surgery, Op technique=operation technique.

*=significant value

Supplementary table 18 - Multiple linear regression assessing whether operative technique significantly explains a difference in lateral flexion on the injured side at 12 months while controlling for age, sex, smoking status, NISS and number of days from trauma to surgery.

R^2^=0.39, *F* (6, 51) = 4.702, *p* <0.001

| Variable | B | SE B | Standardized  Beta | p-value |
| --- | --- | --- | --- | --- |
| Constant  Age  Sex  Smoking  NISS  Op day  Op technique | 23.36  -0.21  1.54  0.21  0.02  0.09  4.52 | 4.05  0.06  1.97  1.10  0.08  0.26  1.67 | -0.53  0.11  0.03  0.04  0.05  0.39 | <0.001*  <0.001*  0.440  0.848  0.780  0.729  0.010* |

Standard multiple linear regression. Dependent variable is lateral flexion on the injured side at 12 months. NISS=New Injury Severity Score, Op day=days from trauma to surgery, Op technique=operation technique.

*=significant value

Supplementary table 19 - Multiple linear regression assessing whether operative technique significantly explains a difference in lateral flexion on the non-injured side at six months while controlling for age, sex, smoking status, NISS and number of days from trauma to surgery.

R^2^=0.42, *F* (6, 53) = 5.720, *p* <0.001

| Variable | B | SE B | Standardized  Beta | p-value |
| --- | --- | --- | --- | --- |
| Constant  Age  Sex  Smoking  NISS  Op day  Op technique | 27.11  -0.21  2.27  0.44  -0.09  0.15  3.35 | 3.47  0.05  1.69  0.94  0.07  0.23  1.43 | -0.58  0.18  -0.06  -0.15  0.08  0.32 | <0.001*  <0.001*  0.186  0.642  0.209  0.518  0.024* |

Standard multiple linear regression. Dependent variable is lateral flexion on the non-injured side at six months. NISS=New Injury Severity Score, Op day=days from trauma to surgery, Op technique=operation technique.

*=significant value

Supplementary table 20 - Multiple linear regression assessing whether operative technique significantly explains a difference in lateral flexion on the non-injured side at 12 months while controlling for age, sex, smoking status, NISS and number of days from trauma to surgery.

R^2^=0.50, *F* (6, 51) = 7.546, *p* <0.001

| Variable | B | SE B | Standardized  Beta | p-value |
| --- | --- | --- | --- | --- |
| Constant  Age  Sex  Smoking  NISS  Op day  Op technique | 23.43  -0.25  2.40  0.48  0.06  0.25  4.45 | 3.29  0.05  1.60  0.89  0.07  0.21  1.36 | -0.69  0.19  0.07  0.10  0.14  0.42 | <0.001*  <0.001*  0.140  0.597  0.362  0.246  0.002* |

Standard multiple linear regression. Dependent variable is lateral flexion on the non-injured side at 12 months. NISS=New Injury Severity Score, Op day=days from trauma to surgery, Op technique=operation technique.

*=significant value

Supplementary table 21 - Multiple linear regression assessing whether operative technique significantly explains a difference in maximum inspiratory pressure at six months while controlling for age, sex, smoking status, NISS and number of days from trauma to surgery.

R^2^=0.53, *F* (6, 66) = 11.366, *p* <0.001

| Variable | B | SE B | Standardized  Beta | p-value |
| --- | --- | --- | --- | --- |
| Constant  Age  Sex  Smoking  NISS  Op day  Op technique | 165.29  -1.12  -27.92  2.35  -0.42  -0.23  8.79 | 17.81  0.23  7.00  4.67  0.30  1.21  7.26 | -0.47  -0.39  0.05  -0.13  -0.02  0.13 | <0.001*  <0.001*  <0.001*  0.617  0.170  0.850  0.231 |

Standard multiple linear regression. Dependent variable is maximum inspiratory pressure at six months. NISS=New Injury Severity Score, Op day=days from trauma to surgery, Op technique=operation technique.

*=significant value

Supplementary table 22 - Multiple linear regression assessing whether operative technique significantly explains a difference in maximum inspiratory pressure at 12 months while controlling for age, sex, smoking status, NISS and number of days from trauma to surgery.

R^2^=0.53, *F* (6, 64) = 11.093, *p* <0.001

| Variable | B | SE B | Standardized  Beta | p-value |
| --- | --- | --- | --- | --- |
| Constant  Age  Sex  Smoking  NISS  Op day  Op technique | 149.44  -1.00  -25.05  3.74  -0.10  -0.02  7.48 | 15.70  0.21  6.17  4.12  0.26  1.07  6.40 | -0.48  -0.40  0.09  -0.04  -0.00  0.13 | <0.001*  <0.001*  <0.001*  0.367  0.704  0.983  0.247 |

Standard multiple linear regression. Dependent variable is maximum inspiratory pressure at 12 months. NISS=New Injury Severity Score, Op day=days from trauma to surgery, Op technique=operation technique.

*=significant value

Supplementary table 23 - Multiple linear regression assessing whether operative technique significantly explains a difference in maximum expiratory pressure at six months while controlling for age, sex, smoking status, NISS and number of days from trauma to surgery.

R^2^=0.45, *F* (6, 66) = 8.170, *p* <0.001

| Variable | B | SE B | Standardized  Beta | p-value |
| --- | --- | --- | --- | --- |
| Constant  Age  Sex  Smoking  NISS  Op day  Op technique | 192.95  -0.97  -34.54  -0.79  -0.38  -0.75  7.82 | 21.96  0.29  8.63  5.76  0.37  1.49  8.95 | -0.35  -0.42  -0.02  -0.11  -0.05  0.10 | <0.001*  0.001*  <0.001*  0.892  0.306  0.617  0.385 |

Standard multiple linear regression. Dependent variable is maximum expiratory pressure at six months. NISS=New Injury Severity Score, Op day=days from trauma to surgery, Op technique=operation technique.

*=significant value

Supplementary table 24 - Multiple linear regression assessing whether operative technique significantly explains a difference in maximum expiratory pressure at 12 months while controlling for age, sex, smoking status, NISS and number of days from trauma to surgery.

R^2^=0.41, *F* (6, 64) = 6.629, *p* <0.001

| Variable | B | SE B | Standardized  Beta | p-value |
| --- | --- | --- | --- | --- |
| Constant  Age  Sex  Smoking  NISS  Op day  Op technique | 183.36  -1.03  -37.72  -1.20  -0.08  0.81  13.10 | 24.99  0.33  9.82  6.55  0.42  1.70  10.18 | -0.35  -0.43  -0.02  -0.02  0.05  0.16 | <0.001*  0.003*  <0.001*  0.855  0.853  0.636  0.203 |

Standard multiple linear regression. Dependent variable is maximum expiratory pressure at 12 months. NISS=New Injury Severity Score, Op day=days from trauma to surgery, Op technique=operation technique.

*=significant value

Supplementary table 25 – Binary logistic regression assessing whether operative technique significantly explains a difference in incidence of winged scapula on the injured side at six months while controlling for age, sex, smoking status, NISS and number of days from trauma to surgery.

χ^2^ (7, n=67) =9.818, *p*=0.199

| Variable | B | SE B | OR | p-value |
| --- | --- | --- | --- | --- |
| Constant  Age  Sex (F)  Smoking (yes)  Smoking (ex)  NISS  Op day  Op technique (muscle-sparing) | -12.63  -0.02  21.53  -16.97  3.62  -0.28  -0.20  -3.13 | 4225.28  0.20  4225.26  6775.71  7.86  0.34  1.10  3.91 | 0.00  0.98  22.3x10^8  0.00  37.47  0.76  0.82  0.04 | 0.998  0.905  0.996  0.998  0.645  0.413  0.856  0.423 |

Binary logistic regression. Dependent variable is incidence of winged scapula on the injured side at six months. NISS=New Injury Severity Score, OR=odds ratio, F=female, Op day=days from trauma to surgery, Op technique=operation technique.

*=significant value

Supplementary table 26 - Multiple linear regression assessing whether operative technique significantly explains a difference in ratio of upper breathing movements on injured side through non-injured side at six months while controlling for age, sex, smoking status, NISS and number of days from trauma to surgery.

| Variable | B | SE B | Standardized  Beta | p-value |
| --- | --- | --- | --- | --- |
| Constant  Age  Sex  Smoking  NISS  Op day  Op technique | 82.23  0.24  -19.09  7.43  -0.16  -0.09  -0.84 | 25.42  0.35  12.38  6.90  0.50  1.65  10.50 | 0.11  -0.26  0.17  -0.05  -0.01  -0.01 | 0.002*  0.497  0.129  0.287  0.749  0.956  0.937 |

R^2^=0.10, *F* (6, 54) = 0.862, *p* =0.529

Standard multiple linear regression. Dependent variable is ratio of upper breathing movements on injured side through non-injured side at six months. Bilateral injuries excluded. NISS=New Injury Severity Score, Op day=days from trauma to surgery, Op technique=operation technique.

*=significant value

Supplementary table 27 - Multiple linear regression assessing whether operative technique significantly explains a difference in ratio of upper breathing movements on injured side through non-injured side at 12 months while controlling for age, sex, smoking status, NISS and number of days from trauma to surgery.

| Variable | B | SE B | Standardized  Beta | p-value |
| --- | --- | --- | --- | --- |
| Constant  Age  Sex  Smoking  NISS  Op day  Op technique | 75.86  0.32  -26.88  1.01  -0.13  0.94  7.67 | 23.97  0.33  11.67  6.50  0.48  1.55  9.90 | 0.16  -0.39  0.03  -0.04  0.10  0.13 | 0.003*  0.331  0.026*  0.877  0.787  0.548  0.443 |

R^2^=0.12, *F* (6, 51) = 0.999, *p* =0.438

Standard multiple linear regression. Dependent variable is ratio of upper breathing movements on injured side through non-injured side at 12 months. Bilateral injuries excluded. NISS=New Injury Severity Score, Op day=days from trauma to surgery, Op technique=operation technique.

*=significant value

Supplementary table 28 - Multiple linear regression assessing whether operative technique significantly explains a difference in ratio of lower breathing movements on injured side through non-injured side at six months while controlling for age, sex, smoking status, NISS and number of days from trauma to surgery.

| Variable | B | SE B | Standardized  Beta | p-value |
| --- | --- | --- | --- | --- |
| Constant  Age  Sex  Smoking  NISS  Op day  Op technique | 122.40  -0.59  24.49  2.89  0.16  -1.97  4.36 | 28.76  0.40  14.01  7.80  0.57  1.86  11.88 | -0.24  0.29  0.06  0.04  -0.16  0.06 | <0.001*  0.144  0.087  0.713  0.776  0.297  0.715 |

R^2^=0.10, *F* (6, 54) = 0.902, *p* =0.502

Standard multiple linear regression. Dependent variable is ratio of lower breathing movements on injured side through non-injured side at six months. Bilateral injuries excluded. NISS=New Injury Severity Score, Op day=days from trauma to surgery, Op technique=operation technique.

*=significant value

Supplementary table 29 - Multiple linear regression assessing whether operative technique significantly explains a difference in ratio of lower breathing movements on injured side through non-injured side at 12 months while controlling for age, sex, smoking status, NISS and number of days from trauma to surgery.

| Variable | B | SE B | Standardized  Beta | p-value |
| --- | --- | --- | --- | --- |
| Constant  Age  Sex  Smoking  NISS  Op day  Op technique | 84.88  0.78  -17.25  -13.99  -1.32  2.28  6.72 | 40.80  0.56  19.87  11.07  0.81  2.64  16.86 | 0.23  -0.15  -0.21  -0.24  0.14  0.07 | 0.043*  0.170  0.390  0.213  0.111  0.393  0.692 |

R^2^=0.13, *F* (6, 51) = 1.114, *p* =0.369

Standard multiple linear regression. Dependent variable is ratio of lower breathing movements on injured side through non-injured side at 12 months. Bilateral injuries excluded. NISS=New Injury Severity Score, Op day=days from trauma to surgery, Op technique=operation technique.

*=significant value

Supplementary table 30 - Multiple linear regression assessing whether operative technique significantly explains a difference in ratio of abdominal breathing movements on injured side through non-injured side at six months while controlling for age, sex, smoking status, NISS and number of days from trauma to surgery.

| Variable | B | SE B | Standardized  Beta | p-value |
| --- | --- | --- | --- | --- |
| Constant  Age  Sex  Smoking  NISS  Op day  Op technique | 90.75  0.25  -6.84  0.04  -0.11  -0.47  -3.10 | 18.51  0.25  9.01  5.02  0.37  1.20  7.65 | 0.16  -0.13  0.00  -0.05  -0.06  -0.07 | <0.001*  0.337  0.452  0.994  0.759  0.699  0.687 |

R^2^=0.03, *F* (6, 54) = 0.245, *p* =0.959

Standard multiple linear regression. Dependent variable is ratio of abdominal breathing movements on injured side through non-injured side at six months. Bilateral injuries excluded. NISS=New Injury Severity Score, Op day=days from trauma to surgery, Op technique=operation technique.

*=significant value

Supplementary table 31 - Multiple linear regression assessing whether operative technique significantly explains a difference in ratio of abdominal breathing movements on injured side through non-injured side at 12 months while controlling for age, sex, smoking status, NISS and number of days from trauma to surgery.

| Variable | B | SE B | Standardized  Beta | p-value |
| --- | --- | --- | --- | --- |
| Constant  Age  Sex  Smoking  NISS  Op day  Op technique | 82.35  -0.06  -12.65  -5.22  0.48  3.43  -2.72 | 23.99  0.33  11.68  6.51  0.48  1.55  9.91 | -0.03  -0.18  -0.13  0.15  0.34  -0.05 | 0.001*  0.861  0.285  0.427  0.317  0.033*  0.785 |

R^2^=0.16, *F* (6, 51) = 1.470, *p* =0.210

Standard multiple linear regression. Dependent variable is ratio of abdominal breathing movements on injured side through non-injured side at 12 months. Bilateral injuries excluded. NISS=New Injury Severity Score, Op day=days from trauma to surgery, Op technique=operation technique.

*=significant value

Supplementary table 32 - Multiple linear regression assessing whether operative technique significantly explains a difference in ratio of maximum upper breathing movements on injured side through non-injured side at six months while controlling for age, sex, smoking status, NISS and number of days from trauma to surgery.

| Variable | B | SE B | Standardized  Beta | p-value |
| --- | --- | --- | --- | --- |
| Constant  Age  Sex  Smoking  NISS  Op day  Op technique | 271.84  -1.76  -1.26  3.76  -0.68  -0.35  18.17 | 56.89  0.78  27.71  15.44  1.13  3.69  23.51 | -0.36  -0.01  0.04  -0.09  -0.01  0.12 | <0.001*  0.029*  0.964  0.809  0.548  0.925  0.443 |

R^2^=0.16, *F* (6, 54) = 1.538, *p* =0.186

Standard multiple linear regression. Dependent variable is ratio of upper breathing movements on injured side through non-injured side at six months. Bilateral injuries excluded. NISS=New Injury Severity Score, Op day=days from trauma to surgery, Op technique=operation technique.

*=significant value

Supplementary table 32 - Multiple linear regression assessing whether operative technique significantly explains a difference in ratio of maximum upper breathing movements on injured side through non-injured side at 12 months while controlling for age, sex, smoking status, NISS and number of days from trauma to surgery.

| Variable | B | SE B | Standardized  Beta | p-value |
| --- | --- | --- | --- | --- |
| Constant  Age  Sex  Smoking  NISS  Op day  Op technique | 108.26  0.36  -7.24  -5.65  -0.91  -0.44  -11.00 | 17.07  0.23  8.31  4.63  0.34  1.11  7.05 | 0.25  -0.14  -0.19  -0.39  -0.06  -0.26 | <0.001*  0.128  0.388  0.229  0.010*  0.695  0.126 |

R^2^=0.18, *F* (6, 51) = 1.630, *p* =0.161

Standard multiple linear regression. Dependent variable is ratio of maximum upper breathing movements on injured side through non-injured side at 12 months. Bilateral injuries excluded. NISS=New Injury Severity Score, Op day=days from trauma to surgery, Op technique=operation technique.

*=significant value

Supplementary table 33 - Multiple linear regression assessing whether operative technique significantly explains a difference in ratio of maximum lower breathing movements on injured side through non-injured side at six months while controlling for age, sex, smoking status, NISS and number of days from trauma to surgery.

| Variable | B | SE B | Standardized  Beta | p-value |
| --- | --- | --- | --- | --- |
| Constant  Age  Sex  Smoking  NISS  Op day  Op technique | 111.00  -0.19  11.75  2.16  0.09  -1.89  -5.41 | 23.91  0.33  11.64  6.49  0.47  1.55  9.88 | -0.10  0.17  0.06  0.03  -0.19  -0.09 | <0.001*  0.568  0.318  0.741  0.850  0.229  0.586 |

R^2^=0.05, *F* (6, 54) = 0.377, *p* =0.890

Standard multiple linear regression. Dependent variable is ratio of maximum lower breathing movements on injured side through non-injured side at six months. Bilateral injuries excluded. NISS=New Injury Severity Score, Op day=days from trauma to surgery, Op technique=operation technique.

*=significant value

Supplementary table 34 - Multiple linear regression assessing whether operative technique significantly explains a difference in ratio of maximum lower breathing movements on injured side through non-injured side at 12 months while controlling for age, sex, smoking status, NISS and number of days from trauma to surgery.

| Variable | B | SE B | Standardized  Beta | p-value |
| --- | --- | --- | --- | --- |
| Constant  Age  Sex  Smoking  NISS  Op day  Op technique | 41.61  0.52  5.28  -9.54  0.09  4.89  -1.24 | 30.16  0.41  14.69  8.18  0.60  1.96  12.46 | 0.20  0.06  -0.18  0.02  0.38  -0.02 | 0.175  0.220  0.721  0.250  0.886  0.016*  0.921 |

R^2^=0.22, *F* (6, 50) = 2.117, *p* =0.070

Standard multiple linear regression. Dependent variable is ratio of maximum lower breathing movements on injured side through non-injured side at 12 months. Bilateral injuries excluded. NISS=New Injury Severity Score, Op day=days from trauma to surgery, Op technique=operation technique.

*=significant value

Supplementary table 35 - Multiple linear regression assessing whether operative technique significantly explains a difference in ratio of maximum abdominal breathing movements on injured side through non-injured side at six months while controlling for age, sex, smoking status, NISS and number of days from trauma to surgery.

| Variable | B | SE B | Standardized  Beta | p-value |
| --- | --- | --- | --- | --- |
| Constant  Age  Sex  Smoking  NISS  Op day  Op technique | 64.40  0.46  -2.97  -0.62  0.25  -0.54  4.43 | 19.65  0.27  9.57  5.33  0.39  1.27  8.12 | 0.28  -0.05  -0.02  0.09  -0.07  0.09 | 0.003*  0.093  0.757  0.908  0.531  0.674  0.588 |

R^2^=0.08, *F* (6, 54) = 0.737, *p* =0.622

Standard multiple linear regression. Dependent variable is ratio of maximum abdominal breathing movements on injured side through non-injured side at six months. Bilateral injuries excluded. NISS=New Injury Severity Score, Op day=days from trauma to surgery, Op technique=operation technique.

*=significant value

Supplementary table 36 - Multiple linear regression assessing whether operative technique significantly explains a difference in ratio of maximum abdominal breathing movements on injured side through non-injured side at 12 months while controlling for age, sex, smoking status, NISS and number of days from trauma to surgery.

| Variable | B | SE B | Standardized  Beta | p-value |
| --- | --- | --- | --- | --- |
| Constant  Age  Sex  Smoking  NISS  Op day  Op technique | 93.81  0.20  -15.52  0.89  -0.25  0.44  -2.33 | 17.72  0.24  8.63  4.81  0.35  1.15  7.32 | 0.14  -0.31  0.03  -0.11  0.06  -0.06 | <0.001*  0.423  0.079  0.854  0.486  0.702  0.752 |

R^2^=0.09, *F* (6, 51) = 0.693, *p* =0.656

Standard multiple linear regression. Dependent variable is ratio of maximum abdominal breathing movements on injured side through non-injured side at 12 months. Bilateral injuries excluded. NISS=New Injury Severity Score, Op day=days from trauma to surgery, Op technique=operation technique.

*=significant value

Supplementary table 37 - Multiple linear regression assessing whether operative technique significantly explains a difference in disability rating index at six months while controlling for age, sex, smoking status, NISS and number of days from trauma to surgery.

| Variable | B | SE B | Standardized  Beta | p-value |
| --- | --- | --- | --- | --- |
| Constant  Age  Sex  Smoking  NISS  Op day  Op technique | -1.37  0.02  0.13  -0.07  0.02  0.01  -0.21 | 0.66  0.01  0.26  0.17  0.01  0.05  0.27 | 0.22  0.07  -0.05  0.17  0.02  -0.11 | 0.041*  0.083  0.604  0.684  0.168  0.865  0.438 |

R^2^=0.11, *F* (6, 74) = 1.416, *p* =0.221

Standard multiple linear regression. Dependent variable is disability rating index at six months. Non-normal data was normalized using Blom’s formula prior to regression analysis. NISS=New Injury Severity Score, Op day=days from trauma to surgery, Op technique=operation technique.

*=significant value

Supplementary table 38 - Multiple linear regression assessing whether operative technique significantly explains a difference in disability rating index at 12 months while controlling for age, sex, smoking status, NISS and number of days from trauma to surgery.

| Variable | B | SE B | Standardized  Beta | p-value |
| --- | --- | --- | --- | --- |
| Constant  Age  Sex  Smoking  NISS  Op day  Op technique | -1.77  0.02  0.16  0.18  0.01  -0.03  0.03 | 0.61  0.01  0.24  0.16  0.01  0.04  0.25 | 0.36  0.08  0.14  0.08  -0.09  0.02 | 0.005*  0.004*  0.519  0.279  0.491  0.490  0.891 |

R^2^=0.18, *F* (6, 73) = 2.501, *p* =0.030

Standard multiple linear regression. Dependent variable is disability rating index at 12 months. Non-normal data was normalized using Blom’s formula prior to regression analysis. NISS=New Injury Severity Score, Op day=days from trauma to surgery, Op technique=operation technique.

*=significant value

Supplementary table 39 - Multiple linear regression assessing whether operative technique significantly explains a difference in Grimby activity scale at 12 months while controlling for age, sex, smoking status, NISS and number of days from trauma to surgery.

| Variable | B | SE B | Standardized  Beta | p-value |
| --- | --- | --- | --- | --- |
| Constant  Age  Sex  Smoking  NISS  Op day  Op technique | 5.51  -0.01  -0.61  -0.31  -0.01  0.03  -0.31 | 0.73  0.01  0.29  0.19  0.01  0.05  0.30 | -0.11  -0.26  -0.21  -0.12  0.07  -0.14 | <0.001*  0.367  0.038*  0.109  0.324  0.560  0.305 |

R^2^=0.14, *F* (6, 73) = 1.885, *p* =0.096

Standard multiple linear regression. Dependent variable is Grimby activity scale at 12 months. NISS=New Injury Severity Score, Op day=days from trauma to surgery, Op technique=operation technique.

*=significant value

Supplementary table 40 - Multiple linear regression assessing whether operative technique significantly explains a difference in EQ5D index at six months while controlling for age, sex, smoking status, NISS and number of days from trauma to surgery.

| Variable | B | SE B | Standardized  Beta | p-value |
| --- | --- | --- | --- | --- |
| Constant  Age  Sex  Smoking  NISS  Op day  Op technique | 0.92  -0.02  0.20  0.01  -0.01  0.01  0.47 | 0.64  0.01  0.25  0.17  0.01  0.04  0.26 | -0.24  0.10  0.01  -0.11  0.04  0.25 | 0.153  0.063  0.436  0.943  0.382  0.786  0.074 |

R^2^=0.13, *F* (6, 74) = 1.625, *p* =0.154

Standard multiple linear regression. Dependent variable is EQ5D index at six months. Non-normal data was normalized using Blom’s formula prior to regression analysis. NISS=New Injury Severity Score, Op day=days from trauma to surgery, Op technique=operation technique.

*=significant value

Supplementary table 41 - Multiple linear regression assessing whether operative technique significantly explains a difference in EQ5D index at 12 months while controlling for age, sex, smoking status, NISS and number of days from trauma to surgery.

| Variable | B | SE B | Standardized  Beta | p-value |
| --- | --- | --- | --- | --- |
| Constant  Age  Sex  Smoking  NISS  Op day  Op technique | 1.04  -0.01  -0.07  -0.18  -0.01  0.01  0.09 | 0.65  0.01  0.25  0.17  0.01  0.04  0.26 | -0.18  -0.04  -0.15  -0.07  -0.03  0.05 | 0.111  0.165  0.789  0.284  0.563  0.828  0.733 |

R^2^=0.09, *F* (6, 73) = 1.039, *p* =0.408

Standard multiple linear regression. Dependent variable is EQ5D index at 12 months. Non-normal data was normalized using Blom’s formula prior to regression analysis. NISS=New Injury Severity Score, Op day=days from trauma to surgery, Op technique=operation technique.

*=significant value

Supplementary table 41 - Multiple linear regression assessing whether operative technique significantly explains a difference in EQ5D VAS at six months while controlling for age, sex, smoking status, NISS and number of days from trauma to surgery.

| Variable | B | SE B | Standardized  Beta | p-value |
| --- | --- | --- | --- | --- |
| Constant  Age  Sex  Smoking  NISS  Op day  Op technique | 0.74  -0.01  0.49  0.09  -0.02  -0.06  0.32 | 0.65  0.01  0.26  0.17  0.01  0.04  0.27 | -0.08  0.24  0.07  -0.18  -0.17  0.16 | 0.257  0.536  0.060  0.589  0.134  0.175  0.237 |

R^2^=0.15, *F* (6, 74) = 2.004, *p* =0.077

Standard multiple linear regression. Dependent variable is EQ5D VAS at six months. Non-normal data was normalized using Blom’s formula prior to regression analysis. NISS=New Injury Severity Score, VAS=visual analogue scale, Op day=days from trauma to surgery, Op technique=operation technique.

*=significant value

Supplementary table 42 - Multiple linear regression assessing whether operative technique significantly explains a difference in EQ5D VAS at 12 months while controlling for age, sex, smoking status, NISS and number of days from trauma to surgery.

| Variable | B | SE B | Standardized  Beta | p-value |
| --- | --- | --- | --- | --- |
| Constant  Age  Sex  Smoking  NISS  Op day  Op technique | 0.60  -0.01  -0.31  -0.15  0.01  -0.01  0.14 | 0.68  0.01  0.27  0.18  0.01  0.05  0.28 | -0.14  -0.15  -0.11  0.05  -0.02  0.07 | 0.377  0.278  0.246  0.416  0.671  0.868  0.622 |

R^2^=0.08, *F* (6, 73) = 0.959, *p* =0.460

Standard multiple linear regression. Dependent variable is EQ5D VAS at 12 months. Non-normal data was normalized using Blom’s formula prior to regression analysis. NISS=New Injury Severity Score, VAS=visual analogue scale, Op day=days from trauma to surgery, Op technique=operation technique.

*=significant value

Supplementary table 43 – Binary logistic regression assessing whether operative technique significantly explains a difference in pain at rest at six months while controlling for age, sex, smoking status, NISS and number of days from trauma to surgery.

χ^2^ (7, n=68) =1.320, *p*=0.988

| Variable | B | SE B | OR | p-value |
| --- | --- | --- | --- | --- |
| Constant  Age  Sex (F)  Smoking (yes)  Smoking (ex)  NISS  Op day  Op technique (muscle-sparing) | -3.31  -0.01  -0.33  -0.04  0.47  0.04  -0.02  -3.31 | 2.74  0.04  1.28  1.45  1.55  0.04  0.20  1.33 | 0.04  0.99  0.97  1.60  1.04  0.98  1.55  1.55 | 0.227  0.760  0.794  0.980  0.762  0.340  0.934  0.743 |

Binary logistic regression. Dependent variable is pain at rest at six months. NISS=New Injury Severity Score, OR=odds ratio, F=female, Op day=days from trauma to surgery, Op technique=operation technique.

*=significant value

Supplementary table 44 – Binary logistic regression assessing whether operative technique significantly explains a difference in pain at rest at 12 months while controlling for age, sex, smoking status, NISS and number of days from trauma to surgery.

χ^2^ (7, n=67) =2.490, *p*=0.928

| Variable | B | SE B | OR | p-value |
| --- | --- | --- | --- | --- |
| Constant  Age  Sex (F)  Smoking (yes)  Smoking (ex)  NISS  Op day  Op technique (muscle-sparing) | -1.34  0.00  -1.27  -0.31  -0.09  -0.03  -0.01  0.49 | 2.50  0.03  1.21  1.25  1.36  0.05  0.20  1.14 | 0.26  1.00  0.28  0.73  0.92  0.97  0.99  1.64 | 0.592  0.953  0.296  0.804  0.950  0.539  0.968  0.665 |

Binary logistic regression. Dependent variable is pain at rest at 12 months. NISS=New Injury Severity Score, OR=odds ratio, F=female, Op day=days from trauma to surgery, Op technique=operation technique.

*=significant value

Supplementary table 45 – Binary logistic regression assessing whether operative technique significantly explains a difference in pain while breathing at six months while controlling for age, sex, smoking status, NISS and number of days from trauma to surgery.

χ^2^ (7, n=68) =4.968 , *p*=0.664

| Variable | B | SE B | OR | p-value |
| --- | --- | --- | --- | --- |
| Constant  Age  Sex (F)  Smoking (yes)  Smoking (ex)  NISS  Op day  Op technique (muscle-sparing) | -0.12  0.01  -1.37  -1.06  -19.79  -0.05  -0.01  -0.74 | 2.67  0.03  1.29  1.28  11836.03  0.06  0.25  1.23 | 0.89  1.01  0.25  0.35  0.00  0.95  0.99  0.48 | 0.964  0.751  0.288  0.408  0.999  0.359  0.968  0.545 |

Binary logistic regression. Dependent variable is pain while breathing at six months. NISS=New Injury Severity Score, OR=odds ratio, F=female, Op day=days from trauma to surgery, Op technique=operation technique.

*=significant value

Supplementary table 46 – Binary logistic regression assessing whether operative technique significantly explains a difference in pain while breathing at 12 months while controlling for age, sex, smoking status, NISS and number of days from trauma to surgery.

χ^2^ (7, n=67) =9.972 , *p*=0.190

| Variable | B | SE B | OR | p-value |
| --- | --- | --- | --- | --- |
| Constant  Age  Sex (F)  Smoking (yes)  Smoking (ex)  NISS  Op day  Op technique (muscle-sparing) | -4.79  0.05  -19.89  1.24  1.17  -0.05  0.09  0.23 | 3.89  0.04  8323.41  1.24  1.41  0.06  0.18  1.41 | 0.01  1.05  0.00  3.45  3.22  0.95  1.09  1.26 | 0.218  0.218  0.998  0.318  0.407  0.382  0.620  0.868 |

Binary logistic regression. Dependent variable is pain while breathing at 12 months. NISS=New Injury Severity Score, OR=odds ratio, F=female, Op day=days from trauma to surgery, Op technique=operation technique.

*=significant value

Supplementary table 47 – Binary logistic regression assessing whether operative technique significantly explains a difference in local tenderness at six months while controlling for age, sex, smoking status, NISS and number of days from trauma to surgery.

χ^2^ (7, n=68) =14.290, *p*=0.046

| Variable | B | SE B | OR | p-value |
| --- | --- | --- | --- | --- |
| Constant  Age  Sex (F)  Smoking (yes)  Smoking (ex)  NISS  Op day  Op technique (muscle-sparing) | -0.34  -0.03  0.08  -1.01  0.13  0.03  0.34  -0.45 | 1.61  0.02  0.71  0.88  0.90  0.03  0.13  0.71 | 0.71  1.08  0.91  0.34  1.14  1.03  1.41  0.64 | 0.833  0.161  0.911  0.216  0.881  0.289  0.011*  0.530 |

Binary logistic regression. Dependent variable is local tenderness at six months. NISS=New Injury Severity Score, OR=odds ratio, F=female, Op day=days from trauma to surgery, Op technique=operation technique.

*=significant value

Supplementary table 48 – Binary logistic regression assessing whether operative technique significantly explains a difference in local tenderness at 12 months while controlling for age, sex, smoking status, NISS and number of days from trauma to surgery.

χ^2^ (7, n=67) =6.429, *p*=0.491

| Variable | B | SE B | OR | p-value |
| --- | --- | --- | --- | --- |
| Constant  Age  Sex (F)  Smoking (yes)  Smoking (ex)  NISS  Op day  Op technique (muscle-sparing) | -2.50  0.02  -0.57  -0.83  0.77  -0.00  0.16  0.07 | 1.77  0.02  0.71  0.91  0.83  0.03  0.12  0.73 | 0.08  1.02  0.57  0.44  2.16  1.00  1.17  0.92 | 0.159  0.391  0.424  0.365  0.356  0.961  0.178  0.923 |

Binary logistic regression. Dependent variable is local tenderness at 12 months. NISS=New Injury Severity Score, OR=odds ratio, F=female, Op day=days from trauma to surgery, Op technique=operation technique.

*=significant value

Supplementary table 49 – Binary logistic regression assessing whether operative technique significantly explains a difference in breathlessness at six months while controlling for age, sex, smoking status, NISS and number of days from trauma to surgery.

χ^2^ (7, n=68) =10.570, *p*=0.159

| Variable | B | SE B | OR | p-value |
| --- | --- | --- | --- | --- |
| Constant  Age  Sex (F)  Smoking (yes)  Smoking (ex)  NISS  Op day  Op technique (muscle-sparing) | -1.57  0.03  -0.28  -1.05  0.64  0.01  -0.31  -0.51 | 1.79  0.03  0.72  0.92  0.97  0.03  0.17  0.79 | 0.21  1.03  0.76  0.35  1.89  1.01  0.74  0.60 | 0.382  0.218  0.696  0.255  0.511  0.764  0.063  0.517 |

Binary logistic regression. Dependent variable is breathlessness at six months. NISS=New Injury Severity Score, OR=odds ratio, F=female, Op day=days from trauma to surgery, Op technique=operation technique.

*=significant value

Supplementary table 50 – Binary logistic regression assessing whether operative technique significantly explains a difference in breathlessness at 12 months while controlling for age, sex, smoking status, NISS and number of days from trauma to surgery.

χ^2^ (7, n=67) =9.013, *p*=0.252

| Variable | B | SE B | OR | p-value |
| --- | --- | --- | --- | --- |
| Constant  Age  Sex (F)  Smoking (yes)  Smoking (ex)  NISS  Op day  Op technique (muscle-sparing) | -3.33  0.06  -0.97  1.09  -0.36  -0.02  -0.31  -0.34 | 2.41  0.03  0.86  0.87  1.28  0.03  0.19  1.00 | 0.04  1.06  0.38  2.96  0.70  0.98  0.73  0.71 | 0.168  0.060  0.257  0.213  0.778  0.584  0.109  0.731 |

Binary logistic regression. Dependent variable is breathlessness at 12 months. NISS=New Injury Severity Score, OR=odds ratio, F=female, Op day=days from trauma to surgery, Op technique=operation technique.

*=significant value

Supplementary table 51 – Binary logistic regression assessing whether operative technique significantly explains a difference in use of pain medication at six months while controlling for age, sex, smoking status, NISS and number of days from trauma to surgery.

χ^2^ (7, n=68) =7.615, *p*=0.368

| Variable | B | SE B | OR | p-value |
| --- | --- | --- | --- | --- |
| Constant  Age  Sex (F)  Smoking (yes)  Smoking (ex)  NISS  Op day  Op technique (muscle-sparing) | -6.68  0.05  -0.26  0.57  -0.64  0.04  0.08  1.21 | 2.62  0.03  0.78  0.90  1.33  0.03  0.13  1.06 | 0.00  1.05  0.77  1.77  0.53  1.04  1.08  3.35 | 0.011*  0.154  0.738  0.523  0.629  0.188  0.567  0.252 |

Binary logistic regression. Dependent variable is use of pain medication at six months. NISS=New Injury Severity Score, OR=odds ratio, F=female, Op day=days from trauma to surgery, Op technique=operation technique.

*=significant value

Supplementary table 52 – Binary logistic regression assessing whether operative technique significantly explains a difference in use of pain medication at 12 months while controlling for age, sex, smoking status, NISS and number of days from trauma to surgery.

χ^2^ (7, n=66) =13.123, *p*=0.069

| Variable | B | SE B | OR | p-value |
| --- | --- | --- | --- | --- |
| Constant  Age  Sex (F)  Smoking (yes)  Smoking (ex)  NISS  Op day  Op technique (muscle-sparing) | -4.03  0.07  0.26  -0.20  -20.14  -0.05  -0.03  -0.46 | 2.92  0.04  0.83  0.98  11541.12  0.05  0.18  1.11 | 0.02  1.07  1.30  0.82  0.00  0.95  0.97  0.63 | 0.167  0.073  0.753  0.838  0.999  0.256  0.874  0.680 |

Binary logistic regression. Dependent variable is use of pain medication at 12 months. NISS=New Injury Severity Score, OR=odds ratio, F=female, Op day=days from trauma to surgery, Op technique=operation technique.

*=significant value

Supplementary table 53 - Multiple linear regression assessing whether operative technique significantly explains a difference in FEV1/FVC at 12 months while controlling for age, sex, smoking status, NISS and number of days from trauma to surgery.

| Variable | B | SE B | Standardized  Beta | p-value |
| --- | --- | --- | --- | --- |
| Constant  Age  Sex  Smoking  NISS  Op day  Op technique | 0.92  -0.00  0.01  -0.10  -0.00  0.00  0.00 | 0.06  0.00  0.02  0.02  0.00  0.00  0.02 | -0.40  0.04  -0.08  -0.06  0.05  0.02 | <0.001*  0.002*  0.751  0.529  0.609  0.719  0.909 |

R^2^=0.17, *F* (6, 73) = 2.203, *p* =0.053

Standard multiple linear regression. Dependent variable is FEV1/FVC at 12 months. FEV1=Forced expiratory volume in one second, FVC=Forced vital capacity, NISS=New Injury Severity Score, VAS=visual analogue scale, Op day=days from trauma to surgery, Op technique=operation technique.

*=significant value
